# Editorial: Motivational interviewing in forensic settings

**DOI:** 10.3389/fpsyg.2026.1848863

**Published:** 2026-04-30

**Authors:** Olga Cunha, Ilana Andretta, Maria Alzira Pimenta Dinis, Sónia Caridade

**Affiliations:** 1 Psychology Research Center (CIPsi), University of Minho, Braga, Portugal; 2 University of the Rio dos Sinos Valley, São Leopoldo, Brazil; 3 Fernando Pessoa Research, Innovation and Development Institute (FP-I3ID), University Fernando Pessoa (UFP), Porto, Portugal

**Keywords:** addiction treatment, criminal justice settings, forensic settings, justice-involved clients, motivational interviewing techniques, offenders' rehabilitation, readiness to change, resistance to change

Motivational interviewing (MI) is a collaborative, goal-oriented, and person-centered approach designed to strengthen an individual's own motivation and commitment to change by eliciting and exploring personally meaningful reasons for change (Miller and Rollnick, 2023; Miller, 2023). Rather than relying on confrontation or persuasion, MI works through empathy, partnership, and the strategic evocation of change talk, which makes it especially relevant when ambivalence and resistance to change are prominent (Forsberg et al., 2025; Lim et al., 2025). This relevance is particularly evident in forensic and correctional settings, where many justice-involved individuals enter treatment, supervision, or rehabilitation under legal pressure rather than through fully voluntary help-seeking, and where motivation to change is closely tied to treatment engagement and clinical progress (Simpson and Penney, 2025; Zhao et al., 2025). In these contexts, MI offers an especially valuable framework because it allows practitioners to address resistance without escalating discord, while helping clients connect change goals to their own values, concerns, and future aspirations (Brockbank and Atkinson, 2026; Pinto e Silva et al., 2025b).

MI has been used in forensic practice to promote individuals' engagement in supervision and in intervention or rehabilitation processes, as well as to elicit and explore their own motivations regarding change goals related to offending behavior, substance use, and other criminogenic targets (Pinto e Silva et al., 2023, 2025b). Recent evidence suggests that interventions incorporating MI are associated with higher session attendance and lower dropout among justice-involved people (Cunha et al., 2024b; Pinto e Silva et al., 2023), and the most recent broad meta-analytic synthesis also found evidence favoring MI for official recidivism outcomes, although effects vary across populations, offenses, and intervention types (Pinto e Silva et al., 2025b). More focused recent studies have likewise reported gains in motivation, treatment adherence, and related outcomes in offender programs, including intimate partner violence interventions (Cunha and Caridade, 2023; Cunha et al., 2024a; Pinto e Silva et al., 2025a), while a recent forensic mental health study found that a motivational preparatory program increased motivation for change and subsequent engagement in treatment and programming (Moulden et al., 2026).

Accordingly, the growing interest in MI with justice-involved individuals is better described as reflecting an expanding and increasingly supportive evidence base, rather than a uniform proof of efficacy across all forensic populations (van der Baan et al., 2024).

This is important because contemporary forensic and correctional services are increasingly expected to move beyond simple control and sanction and toward rehabilitation, recovery, and reintegration-oriented care (Simpson and Penney, 2025; Zhao et al., 2025). Yet coercive conditions, power imbalances, and implementation barriers still make it difficult to engage individuals in change processes across criminally relevant areas such as substance use, treatment participation, and broader responsivity to intervention (Brockbank and Atkinson, 2026; Zhao et al., 2025). In this regard, MI has several advantages in forensic work: it is an evidence-informed method for improving attendance and reducing dropout, it can strengthen motivation and readiness for change, and it offers a practical way to respond to reluctance while preserving a constructive working alliance and supporting subsequent treatment engagement (Brockbank and Atkinson, 2026; Cunha et al., 2024b; Moulden et al., 2026; Pinto e Silva et al., 2025b).

The present Research Topic provides a timely framework for reconsidering the place of MI in forensic practice, where the challenge of promoting change is frequently shaped by legal mandate, low readiness, and variable engagement (Pinto e Silva et al., 2025b; Zhao et al., 2025). Recent evidence suggests that MI-informed interventions may be particularly valuable in justice settings because they are associated with stronger session attendance and lower dropout, while also contributing to more constructive rehabilitative processes among justice-involved individuals (Cunha et al., 2024b; Pinto e Silva et al., 2025b). In parallel, newer findings from forensic mental health indicate that motivationally oriented preparatory interventions can strengthen motivation for change and increase subsequent participation in treatment and programming, reinforcing the importance of attending to readiness before and alongside formal intervention (Moulden et al., 2026). At the same time, emerging work with detained youth shows that the impact of MI may depend on developmental and contextual fit, suggesting that its application in forensic settings must remain sensitive to population-specific needs rather than assumed to be uniformly effective across groups (van der Baan et al., 2024). Read in this way, the present Topic highlights not only the clinical relevance of MI for supporting engagement and rehabilitation, but also the importance of understanding its implementation as an organizational and practice-based process shaped by training, resources, workplace context, and treatment fidelity (Brockbank and Atkinson, 2026).

Against this background, the contributions brought together in this Research Topic examine psychological frameworks and professional practices across correctional and rehabilitative settings, underscoring the relevance of MI principles.

Tafrate et al. develop and provide the initial validation of the Response Style Screening Questionnaire (RSSQ), a brief self-report tool designed to assess MI practice orientation among criminal justice practitioners. MI has become an important communication approach in correctional and probation contexts to enhance engagement and promote behavioral change among justice-involved individuals. The authors conducted three empirical studies involving criminal justice practitioners, including probation officers, to examine the questionnaire's factor structure, reliability and validity. Exploratory and confirmatory factor analyses identified an 18-item instrument organized into four response styles: two MI-consistent styles (Eliciting and Change Talk) and two MI-inconsistent styles (Confrontational and Sustain Talk). The results demonstrate acceptable psychometric properties and show that MI-consistent styles are associated with practitioner orientations emphasizing behavioral change and collaborative engagement with clients. The authors argue that the RSSQ provides a practical and efficient screening tool that can help agencies assess practitioners' communication styles, evaluate training outcomes and support the implementation of evidence-based correctional practices.

Maldonado and Dykerman examine the linguistic structure of recidivism treatment manuals used by public offender counselors to better understand how language reflects and shapes correctional rehabilitation practices. Using a corpus-based linguistic analysis, the authors analyse the textual content of two widely used cognitive-behavioral intervention manuals, Cognitive Behavioral Interventions–Core Adult (CBI-CA) and Thinking for a Change (T4C), to identify recurring patterns in vocabulary and thematic emphasis. The findings reveal distinct linguistic profiles between the manuals, with CBI-CA emphasizing instructional and session-based terminology such as “module,” “session,” and “worksheet,” while T4C more frequently connects crime-related concepts with broader justice and systemic themes. These differences suggest that treatment manuals may implicitly frame offender rehabilitation in different ways, potentially influencing how counselors structure interventions and engage clients. The study highlights the value of corpus-based methods for examining correctional treatment materials and provides insights into how language can shape the delivery of evidence-based rehabilitation programs in offender counseling.

Lee and Park explore the emotional experiences of Korean correctional officers who respond to incidents of self-harm among incarcerated individuals. Using a qualitative research design, the authors conducted in-depth interviews with 15 correctional officers and analyzed the data using Colaizzi's phenomenological method. The findings reveal that officers experience complex emotional reactions including shock, fear, empathy, moral conflict and emotional exhaustion when managing self-harm incidents in prisons. Participants described the psychological burden associated with repeated exposure to such events, alongside feelings of professional responsibility and concern for inmate wellbeing. The study also identifies coping strategies used by officers, such as emotional distancing and peer support, though these strategies often provide only partial relief from stress. The authors emphasize the need for institutional support systems, psychological counseling and training programmes to mitigate occupational stress among correctional staff and improve prison mental health management.

Morlat and Alison examine the concept of childhood poly-victimization and its implications for investigative interviewing with child victims. Using a systematic review of empirical studies published since 2007, the authors analyse how poly-victimization has been conceptualized and measured across research involving populations under 18 years of age. The findings reveal considerable variation in definitions, measurement approaches and thresholds used to identify poly-victimization, including differences in the number of victimization types required, time frames considered and the inclusion of mental health consequences. This lack of conceptual consistency complicates the identification of poly-victims and may hinder effective responses within forensic and law-enforcement contexts. To address this Research Topic, the authors propose a clearer definition of childhood poly-victimization and introduce a decision-tree framework intended to assist practitioners during child investigative interviews. The study highlights the importance of rapport-based interviewing and coherent conceptual frameworks to improve the identification of abuse and better safeguard vulnerable children.

The articles presented above explore psychological frameworks and professional practices across correctional, rehabilitation and safeguarding contexts. They highlight how evidence-based tools, intervention materials and practitioner experiences contribute to improving institutional responses to complex behavioral and social challenges. The contributions include the development of a validated screening instrument designed to assess MI communication styles among criminal justice practitioners, supporting the implementation of collaborative approaches to behavioral change. Complementary research analyses the linguistic structure of recidivism treatment manuals, demonstrating how the language used in rehabilitation materials reflects different conceptualisations of offender change processes. Another study examines the emotional experiences of correctional officers responding to incidents of inmate self-harm, revealing the psychological burden associated with repeated exposure to such events and the coping strategies employed by staff. Finally, a systematic review clarifies the concept of childhood poly-victimization and proposes a decision-tree framework to support practitioners in identifying abuse during child investigative interviews. These contributions highlight the relevance of MI principles, practitioner communication skills and evidence-based frameworks for enhancing engagement, rehabilitation processes and investigative practices within forensic and criminal justice settings.

## References

[B1] Brockbank C. Atkinson C. (2026). Motivational interviewing in service delivery: a systematic literature review of organisational factors affecting implementation. J. Ment. Health Train. Educ. Pract. 21, 33–47. doi: 10.1108/JMHTEP-10-2024-0106

[B2] Cunha O. Almeida T. C. Gonçalves R. A Caridade S. (2024a). Effectiveness of the motivational interviewing techniques with perpetrators of intimate partner violence: a non-randomized clinical trial. J. Aggress. Maltreatment Trauma 33, 291–310. doi: 10.1080/10926771.2023.2189043

[B3] Cunha O. Caridade S. (2023). “Batterer intervention program (BIP)”, in Encyclopedia of Domestic Violence, ed. T. K. Shackelford (Cham: Springer), 1–11. doi: 10.1007/978-3-030-85493-5_88-1

[B4] Cunha O. Pedrosa J. Silva Pereira B. Caridade S. de Castro Rodrigues A. Braga T. (2024b). Intervention program dropout among perpetrators of intimate partner violence: a meta-analysis of correlated variables. Trauma Violence Abuse 25, 2735–2751. doi: 10.1177/15248380231224036 38323403

[B5] Forsberg L. G. Forsberg L. Miller W. R. (2025). What's wrong with motivational interviewing? I. Theoretical and methodological critiques. Behav. Cogn. Psychother 53, 264–274. doi: 10.1017/S1352465825000086 40665863

[B6] Lim K. Jung Y.-C. Kim B.-H. (2025). Evaluating motivational interview quality using large language models and hidden Markov models. BMC Psychiatry 25:908. doi: 10.1186/s12888-025-07391-1 41034852 PMC12487504

[B7] Miller W. R. (2023). The evolution of motivational interviewing. Behav. Cogn. Psychother. 51, 616–632. doi: 10.1017/S1352465822000431 37170826

[B8] Miller W. R. Rollnick S. (2023). Motivational Interviewing: Helping People Change and Grow. New York, NY: The Guildford Press.

[B9] Moulden H. M. Matthew S. A. Chaimowitz G. (2026). Enhancing pre-treatment motivation improves forensic mental health outcomes. Int. J. Offender Ther. Comp. Criminol. 70, 150–166. doi: 10.1177/0306624X241228229 38314713 PMC12756526

[B10] Pinto e Silva T. Cunha O. Caridade S. (2023). Motivational interview techniques and the effectiveness of intervention programs with perpetrators of intimate partner violence: a systematic review. Trauma Violence Abuse 24, 2691–2710. doi: 10.1177/15248380221111472 35793513 PMC10486163

[B11] Pinto e Silva T. Dinis M. A. P. Pedrosa e Sousa H. F. Cunha O. Caridade S. (2025a). Short motivational program for perpetrators of intimate partner violence: a feasibility study. J. Police Crim. Psychol. 40, 719–731. doi: 10.1007/s11896-024-09680-z

[B12] Pinto e Silva T. Gouveia C. Santirso F. A. Cunha O. Caridade S. (2025b). Effectiveness of motivational interviewing with justice-involved people: a systematic review and meta-analysis. Psychosoc. Interv. 34, 89–102. doi: 10.5093/pi2025a8 40405915 PMC12097221

[B13] Simpson A. I. F. Penney S. R. (2025). International developments in the provision of recovery-oriented care in forensic mental health services. World Psychiatry 24, 269–271. doi: 10.1002/wps.21320 40371772 PMC12079520

[B14] van der Baan H. S. Collot D'Escury-Koenigs A. L. Grasman R. P. P. P. Schippers G. M. Wiers R. W. (2024). Combining motivational interviewing and cognitive bias modification training for substances in detained youth. JAACAP Open 2, 301–310. doi: 10.1016/j.jaacop.2024.08.003 39697389 PMC11650685

[B15] Zhao J. Junes S. Canning C. Jull J. Mishra A. Waddell A. . (2025). Patient engagement in forensic mental health care: a scoping review. BMJ Ment. Health 28:e301678. doi: 10.1136/bmjment-2025-301678 41027678 PMC12481395

